# From the Solution Processing of Hydrophilic Molecules to Polymer-Phthalocyanine Hybrid Materials for Ammonia Sensing in High Humidity Atmospheres

**DOI:** 10.3390/s140813476

**Published:** 2014-07-24

**Authors:** Pierre Gaudillat, Florian Jurin, Boris Lakard, Cédric Buron, Jean-Moïse Suisse, Marcel Bouvet

**Affiliations:** 1 Institut de Chimie Moléculaire de l'Université de Bourgogne, UMR CNRS 6302, Université de Bourgogne, 9 Avenue Alain Savary, 21078 Dijon Cedex, France; E-Mails: pierre.gaudillat@u-bourgogne.fr (P.G.); jean.suisse@u-bourgogne.fr (J.-M.S.); 2 Institut UTINAM, UMR CNRS 6213, Université de Franche-Comté, 16 Route de Gray, 25030 Besançon Cedex, France; E-Mails: florian.jurin@univ-fcomte.fr (F.J.); boris.lakard@univ-fcomte.fr (B.L.)

**Keywords:** solution processing, polyaniline, phthalocyanine, hybrid material, layer-by-layer, ammonia, relative humidity, conductometric sensor

## Abstract

We have prepared different hybrid polymer-phthalocyanine materials by solution processing, starting from two sulfonated phthalocyanines, s-CoPc and CuTsPc, and polyvinylpyrrolidone (PVP), polyethylene glycol (PEG), poly(acrylic acid-co-acrylamide) (PAA-AM), poly(diallyldimethylammonium chloride) (PDDA) and polyaniline (PANI) as polymers. We also studied the response to ammonia (NH_3_) of resistors prepared from these sensing materials. The solvent casted films, prepared from s-CoPc and PVP, PEG and PAA-AM, were highly insulating and very sensitive to the relative humidity (RH) variation. The incorporation of s-CoPc in PDDA by means of layer-by-layer (LBL) technique allowed to stabilize the film, but was too insulating to be interesting. We also prepared PANI-CuTsPc hybrid films by LBL technique. It allowed a regular deposition as evidenced by the linear increase of the absorbance at 688 nm as a function of the number of bilayers. The sensitivity to ammonia (NH_3_) of PANi-CuTsPc resistors was very high compared to that of individual materials, giving up to 80% of current decrease when exposed to 30 ppm NH_3_. Contrarily to what happens with neutral polymers, in PANI, CuTsPc was stabilized by strong electrostatic interactions, leading to a stable response to NH_3_, whatever the relative humidity in the range 10%–70%. Thus, the synergy of PANI with ionic macrocycles used as counteranions combined with their simple aqueous solution processing opens the way to the development of new gas sensors capable of operating in real world conditions.

## 1. Introduction

In the field of materials for gas sensing, the historical non-stoichiometric tin oxide, SnO_2-x_, first patented by Taguchi fifty years ago [1] and commercialized by the Figaro Company, is still widely used and studied. Beside SnO_2−x_, used as the basis of millions of sensors on the market per year, many other metal oxides have been studied, like WO_3−x_, ZnO_1−x_, In_2_O_3−x_, or V_2_O_5−x_ [2], all operating between 200 and 500 °C [3,4]. Since the first studies, various preparation methods, in 3D, 2D and even 1D, have been proposed using pure oxides [5,6], metal-doped materials [7,8] or materials decorated by nanoparticles [9,10]. The drift of their structure and morphology leads to the lack of reproducibility of the obtained materials. In all cases, their electronic properties and their sensitivity to gases arise from their non-stoichiometry and from the oxygen contained on their surfaces [11–13].

However, a strong demand exists for gas sensors operating at lower temperature and prepared with low energy processes. Thus molecular materials [14–16], including polymeric materials that can be often deposited by solution processing, are good candidates. Another interest in molecular materials results from the variability of the accessible materials, including hybrid organic-inorganic materials, and materials combining small molecules with carbonaceous compounds [17–21] or polymers [22–25]. Both the composition of the hybrid material and the parameters of the solution processing stage can be used to tune the characteristics of the obtained materials, e.g., morphology, roughness and specific surface, hydrophilicity or hydrophobicity and eventually their optical and electronic properties. Thus, emerging sensor production technologies have become available [26–29], such as ink-jet printing, which is particularly suitable for large areas and low cost processes. Besides, electrodeposition presents clear advantages [30,31], for example when different materials need to be deposited on different electrodes located on the same substrate.

The first aim of this work was to show that solution processing offers a convenient method for the preparation of sensing layers. The materials were chosen because it was known on the one hand that phthalocyanines are interesting molecular materials for conductometric sensors, see [14,15], and on the other hand that polymer—phthalocyanine hybrid materials were already demonstrated to be less cristalline and more efficient for gas sensing than pure phthalocyanines, with better kinetics [24]. Thus we reported therein different solution processing approaches with sulfonated phthalocyanines. The second goal was to find a method to obtain a conductometric sensor capable of detecting ammonia (NH_3_) and stable in a wide range of relative humidity (RH). Indeed, a key point for most of the applications of ammonia sensors is their behavior under humidity. NH_3_ is encountered frequently in industry because of its use as a freezing gas and as a raw material for fertilizers [32]. As a result, it is important to monitor NH_3_ concentrations in air as a safety measure to protect industrial employees. In order to do so, European air quality labor legislation for NH_3_ sets a daily exposure limit at 20 ppm. Many detectors that cover this range are available for sale. However, their main drawback is their lack of selectivity towards NH_3_, in particular within an industrial environment where the humidity level can vary in broad range and modify the sensor response. In the literature, when the effect of the humidity is studied, either it is studied independently of NH_3_, in a separate experiment, so the synergy between NH_3_ and water is not taken into account [33], or the response to NH_3_ is studied under water, but only at a particular RH and not in a broad range. Thus, the response of a PANI resistor was studied in a broad range of RH, but only at 100 ppm NH_3_, moreover with exposure during 4 h [34]. PEDOT/PSS resistors were studied at 7% and 55% of RH, but at one very high NH_3_ concentration (1%) and also at 0.7 ppm but at only one very low RH value, namely 12.5% [35]. In both cases it was very far from a study in a broad humidity range, so the present study was important for matching sensors and applications [32]. Additionally, another advantage of sulfonated phthalocyanines is to fit into sustainable development in the preparation of the devices, by means of solution processing, using water as a solvent, for a low environmental impact and without using bulky and costly hardware. This was an ambitious aim since water-soluble materials exhibit generally high hydrophilicity and hygroscopicity. In one of our previews works [36], a sulfonated water-soluble phthalocyanine (s-CoPc) sensor prepared by solvent cast in water was used for ammonia sensing. The solvent cast films were not stable under high humidity exposure due to the high hygroscopicity of the material; the molecules did not bind strongly enough to the substrate and moved on its surface when the sensor was exposed to high humidity atmospheres. Moreover, water molecules going through the phthalocyanine layer made possible the reduction of the ITO electrodes, under an applied potential of +3 V. To improve the stability of the layers, two possibilities were envisioned. The first possibility was to add different hydrophilic polymers, such as polyvinylpyrrolidone (PVP), polyethylene glycol (PEG) and poly(acrylic acid-co-acrylamide) (PAA-AM), selected for their ammonia sensing or water stability, as reported in literature [37–39], to s-CoPc. These mixed solutions were also used by solvent-casting from water solutions. Another possibility was the Layer-by-Layer (LBL) material processing [40,41], which induces strong interactions between the substrate and two polyelectrolytes of opposite charges, and should be a good alternative to using hydrophilic materials for applications that require stability at high humidity levels and for a long enough time. This method was applied to electrochemical sensors [40,41], but is not commonly used for resistors, despite of its crucial advantages. It can also be performed in water solutions without using any organic solvent, as it is often the case for solvent casting, and can be easily automated to produce a high number of samples for industrial applications. When the experimental conditions are established, the samples can be produced with high reproducibility too. Poly(diallyldimethylammonium chloride) (PDDA) is one of the most used cationic polyelectrolytes [42,43]. This polymer does not conduct the current, but could be added to the sulfonated phthalocyanine as anionic counterpart to prepare a resistor by the LBL technique for ammonia sensing. PANI is a highly conductive polymer used for many applications [34,35,44–46]. It exists in different forms depending on the synthetic route [47], and has been used as a cationic polyelectrolyte for LBL processing with tetrasulfonated phthalocyanines (CuTsPc, NiTsPc and FeTsPc) for electrochemical sensing applications [48]. In the present work, different hybrid polymer-phthalocyanine material–based resistors were prepared using two different sulfonated phthalocyanines. Their stability under humidity and their response to ammonia in the ppm range, over a broad humidity range, were also studied.

## 2. Experimental Section

### 2.1. Electrical Measurements

All fundamental electrical and sensor measurements were performed at room temperature, using a 6517B Keithley electrometer equipped with an embedded DC voltage supply. The electrometer was controlled through a home-made software relying on a GPIB (IEEE488.2) bus connection for data communication. Current-voltage (I-V) curves were registered with voltage values ranges depending on the devices while taking care to start and finish at 0 V bias, in order to avoid any irreversible polarization effect, except for gas sensing experiments. Usually our resistors are polarized with a constant bias of +1 or +3 V. With humidity exposure and hydrophilic and hygroscopic materials, +1 V was selected to prevent the electrodes reduction by water into the film. The solvent casted devices were used with a particular protocol to minimize the protonic conductivity: The current measured was the average of the absolute current measured at +3 V and −3 V. All experiments were performed in dark conditions to avoid any photoelectric effects that may have occurred otherwise.

### 2.2. Gas Sources for Sensing Experiments

**Figure 1.**
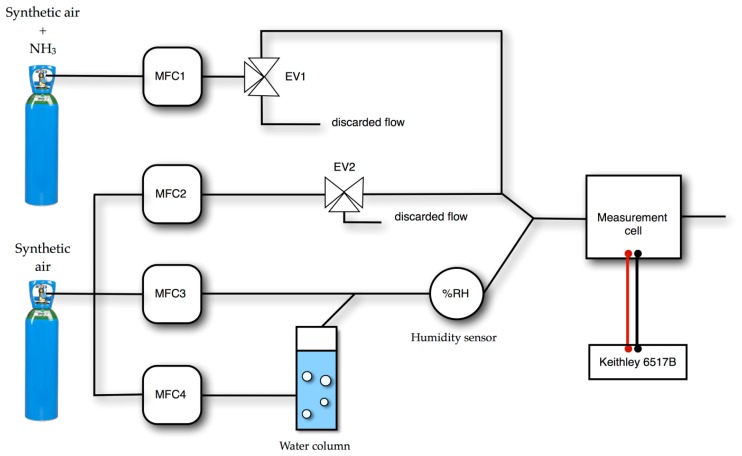
Scheme of the ammonia workbench.

The ammonia gas from a standard cylinder (1000 ppm in synthetic air, from Air Liquide, Paris, France) was diluted with dry synthetic air using mass flow controllers (total flow: 0.5 to 0.55 NL·min^−1^ depending on ammonia concentration) in order to achieve stable, controlled and adjustable ammonia concentrations from 10 to 90 ppm. The experimental set-up (Figure 1) used for electrical measurements and gas exposure was identical to the previously reported one [30], one dry line allowing the control of the NH_3_ flow and another one allowing the control of the relative humidity, by means of a water column and a humidity sensor, from Vaisala (Helsinki, Finland). The response to ammonia was determined after alternation of exposure and recovery periods. The exposure and recovery times were fixed at 1 and 4 min, respectively, unless otherwise indicated. The interest of such a dynamic process rather than a static process is that it avoids the irreversible occupation of sites as it occurs when the material is exposed over very long durations [15,49]. All the experiments were carried out at a room temperature in the range 18–22 °C.

### 2.3. Materials and Chemicals

**Figure 2.**
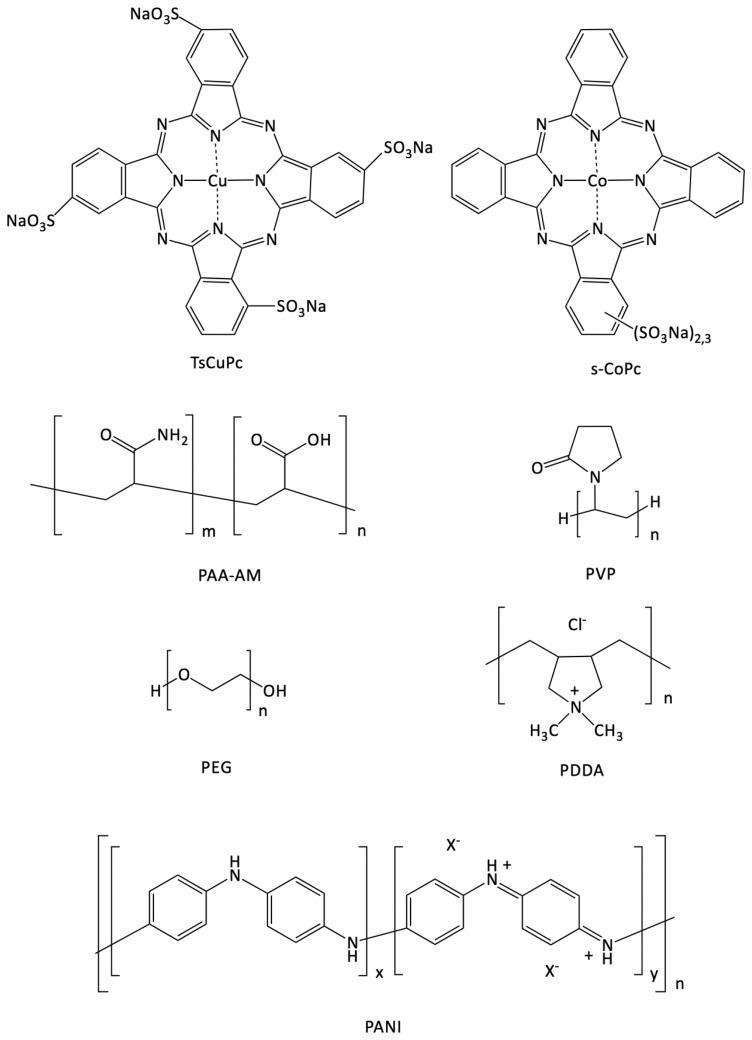
Structural formulas of compounds used in this study.

The sulfonated cobalt phthalocyanine s-CoPc is an industrial product, a mixture of *n*-sulfonated CoPc, known as Co[(SO_3_Na)_2,3_Pc], supplied by the Europthal Company (Cavaillon, France) as additive 8020 (Figure 2). s-CoPc solutions were prepared at a concentration of 7.0 × 10^−1^ and 1 mg·mL^−1^ for solvent casting with a polymer and for LBL processing with PDDA, respectively. The polymer was added to the s-CoPc solutions (PAA-AM, PEG and PVP) to obtain 80%/20% (w/w) solutions for solvent cast processing. PAA-AM (M_w_ = 5,000,000 Da), PEG (M_w_ = 1450) and PVP (M_w_ = 40,000) were purchased from Aldrich Co. (Wyoming, Il, US). The polymer concentrations in the solvent casted solutions prepared with distilled water were respectively: [PAA] = 2.37 × 10^−1^ mg·mL^−1^, [PEG] = 1.71 × 10^−1^ mg·mL^−1^ and [PVP] = 1.83 × 10^−1^ mg·mL^−1^. The solvent casted solutions concentrations were calculated to obtain approximately 100 nm—thick films, as a rough estimation from the surface and the density of the materials. PDDA was purchased from Aldrich Co. (M_w_ = 200,000–350,000) and diluted in distilled water at a concentration of 20 mM.

Tetrasulfonated copper phthalocyanine CuTsPc was purchased from Sigma-Aldrich Co. (dye content, 85%) and dissolved in water at a concentration of 10 mM and pH adjusted to 2.8 with HCl. This solution was used with PANI for LBL processing. PANI was synthesized as following: 0.2 g of aniline monomer was introduced in 50 mL of water and pH was adjusted at 2.4 with concentrated HCl (37 wt %). Solution was then stirred during 1 h. Polymerization was initiated by addition of 0.25 g of ammonium persulfate (APS). After 16 h of reaction at room temperature, three cycles of centrifugation/redispersion in water were carried out to remove APS and residual unpolymerized material. PANI solutions were finally stored in dark conditions to avoid any degradation. All our devices were built by successive deposition of the corresponding molecular materials onto ITO interdigitated electrodes separated by 75 μm on 1 cm × 1 cm glass substrates.

### 2.4. s-CoPc/Polymer Solvent Casted Films

The solvent casted films were prepared using the solutions described above. For each sample with s-CoPc and PAA-AM, PEG and PVP, named E1, E2 and E3, respectively, 10 drops of the solvent cast solutions were deposited onto the substrate. The substrates were heated at 50 °C in a closed desiccator, to increase the homogeneity of the films [23].

I-V characteristics were performed on E1, E2 and E3, from −10 to +10 V. Depending on the atmospheric conditions in the laboratory during the experiments, the measured current varied a lot and the experiments were not usable. To investigate more deeply this effect, humidity exposures from 0% RH to 80% RH were performed by applying a potential of +3 V, −3 V and 0 V as described before.

**Figure 3.**
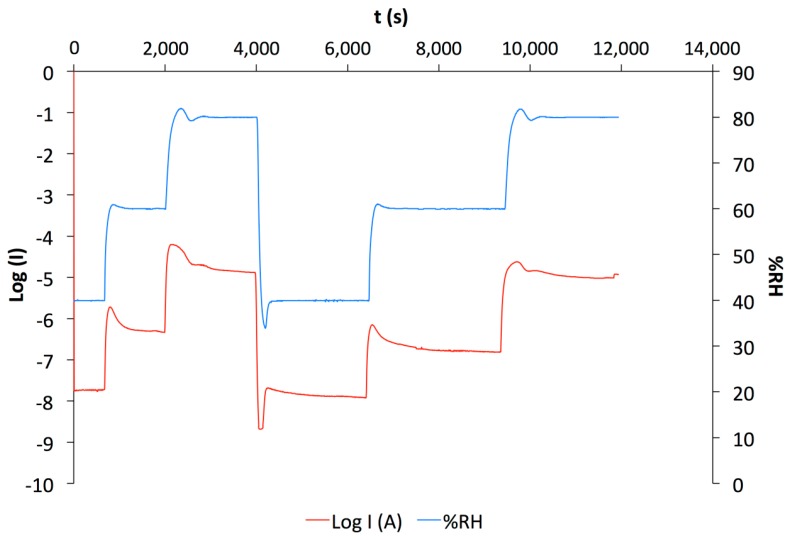
Humidity exposure experiment performed on s-CoPc/PEG sample (E2).

The samples were insulators at 0% RH and showed a huge current increase from 1 × 10^−10^ A at low humidity up to 5.0 × 10^−5^ A at high humidity (Figure 3). The sensors exhibited a high response to the ambient humidity increasing the current by 5 orders of magnitude. These results could be predicted with hydrophilic materials added to s-CoPc that is also highly sensitive to humidity. However, knowing that these huge current increases occur, the humidity can be predicted using the current baseline and log(I) instead of I, as illustrated by Figure 3. Varying the humidity, the log of the current varies but comes back approximately to the same value for each relative humidity.

**Figure 4.**
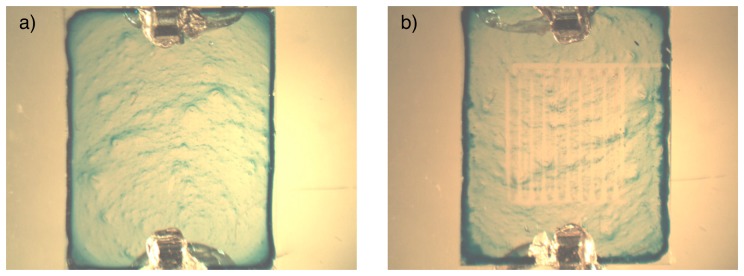
PVP resistor before humidity exposure (**a**); and after humidity exposure (**b**).

The problem of these devices E1, E2 and E3 was the long-term stability. After some hours of high humidity exposure, the films moved on the top of the substrate and leached from the electrodes (Figure 4). Moreover, the high content of water inside the film reduces the ITO electrodes owing to the high applied potential, higher than the oxidation potential of water molecules (Figure 4). To resolve these long-term stability problems, the LBL method was used, using the same ammonia sensitive material, s-CoPc as anionic polyelectrolyte, with PDDA as cationic polyelectrolyte.

### 2.5. s-CoPc/PDDA LBL Films

**Figure 5.**
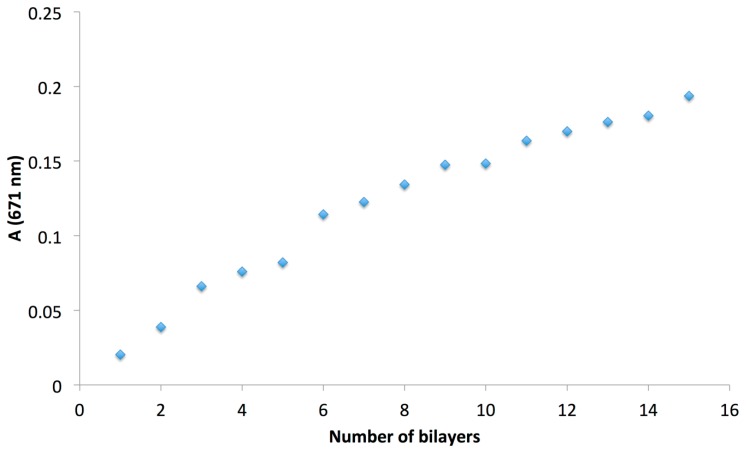
Absorbance measured at 671 nm against the number of PDDA/s-CoPc bilayers.

The substrate was immersed alternatively in the polycation (PDDA) and s-CoPc solution for 5 min each and then rinsed in a distilled water solution for 5 min. The sample was dried in a desiccator at 50 °C after each bilayer (BL) was applied. The process is repeated 15 times to obtain (PDDA/s-CoPc)_15_ sample E4. The growth of the LBL film was monitored by using UV-Vis spectroscopy between 300 to 800 nm where a maximum peak at 671 nm corresponding to the partially sulfonated s-CoPc can be observed (Figure 5). The absorbance increased almost linearly as a function of the number of bilayers, to a maximum of 0.2 a.u. for (PDDA/s-CoPc)_15_. Referring to the literature, this absorbance corresponds approximately to a film thickness of 30–40 nm [33].

**Figure 6.**
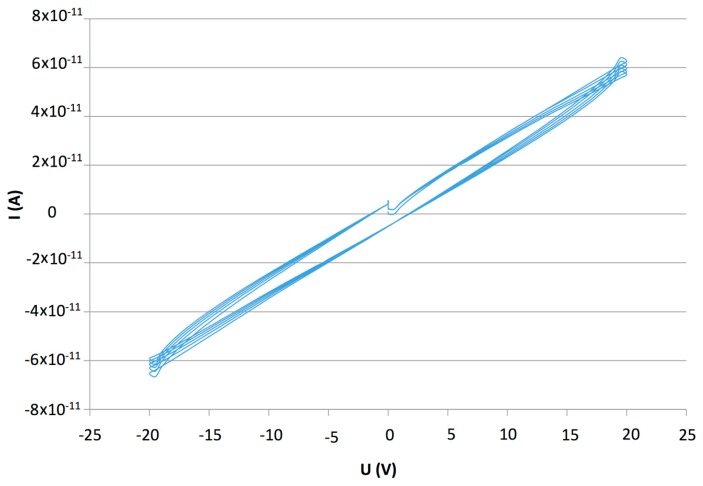
I-V characteristic of the (PDDA/s-CoPc)_15_ device (E4), between −25 V to +25 V.

Multilayer film had the advantage of being stable under high humidity exposure. The electrostatic interactions are stronger than the solvation by water inside the film. For this sample, when polarized at +3 V, no reduction of the electrodes by water occurred. These advantages are crucial to our devices. The LBL deposition method allows the use of highly hydrophilic molecules for sensing applications in wet atmosphere. The problem with this LBL film is the insulating character of the PDDA polymer that added to s-CoPc makes the film highly insulating , as shown in Figure 6.

s-CoPc is a p-type semiconductor on which ammonia induces a current decrease and the higher measured current is lower than 1 × 10^−10^ A at +25 V, which is too low to obtain repeatable and measurable current values. This problem can be solved by using a more conductive cationic polyelectrolyte such as PANI. CuTsPc was used instead of s-CoPc to improve the film growth maximizing the negative charges in the phthalocyanine molecule.

### 2.6. CuTsPc/PANI LBL Films

#### 2.6.1. Material Processing

**Figure 7.**
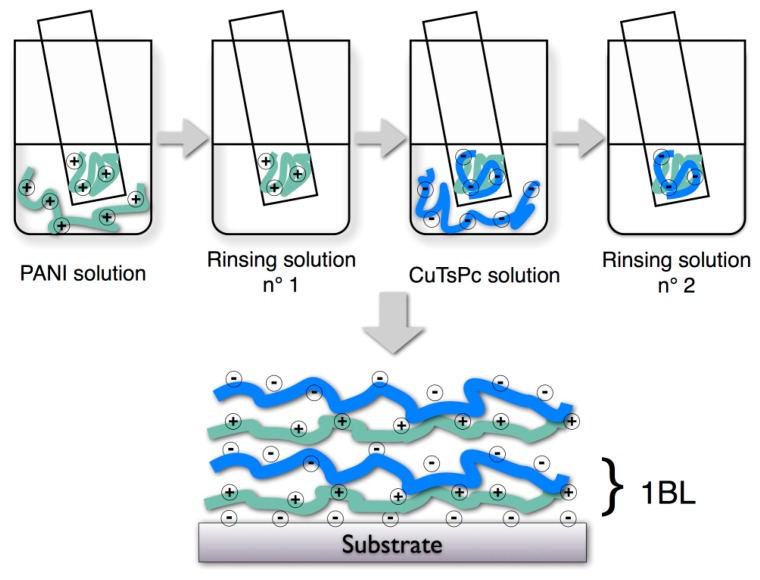
Scheme of the LBL process for PANI/CuTsPc films.

The PANI solution was diluted with distilled water to a concentration of 0.3 g·L^−1^ and adjusted to pH = 2.8. Two rinsing solutions, n° 1 and 2, were prepared with distilled water at pH = 2.8. The ITO/glass substrates were immersed successively in the PANI solution for 5 min, rinsed in the solution n° 1 for 5 min, immersed in the CuTsPc solution for 5 min and rinsed 5 min in the solution n° 2 (Figure 7). Then the substrates were dried under air flow.

**Figure 8.**
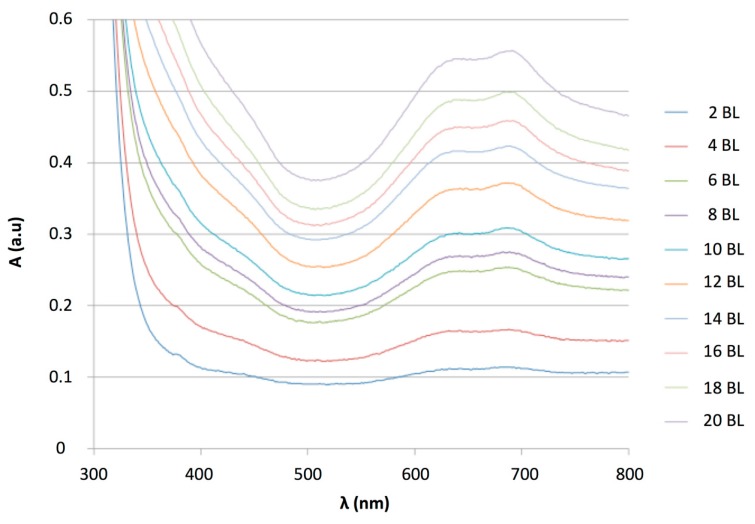
(PANI/CuTsPc)_n_ (E5) absorption spectrum from 300 to 800 nm against the BL number.

**Figure 9.**
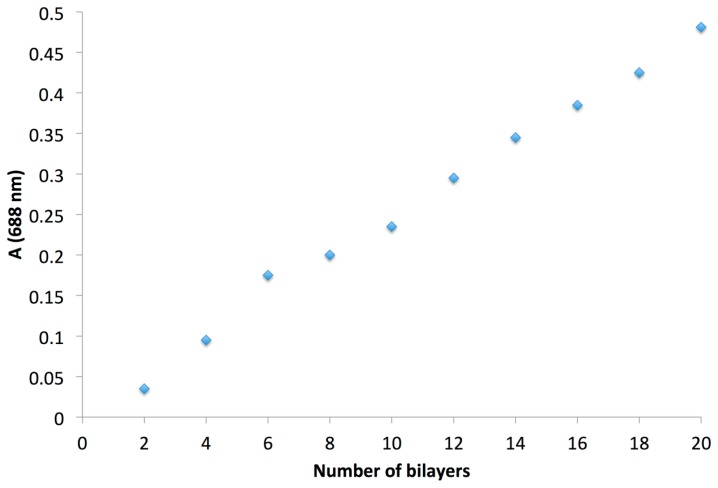
Absorbance measured at 688 nm, with the baseline correction, against the number of (PANI/CuTsPc)_n_ bilayers (E5).

This protocol was repeated 20 times to obtain a 20 bilayer film denoted as sample E5. The growth of the LBL film was monitored by UV-Vis spectroscopy between 300 to 800 nm, with a maximum peak at 688 nm corresponding to CuTsPc (Figure 8). It is worth noting that the PANI contributes also to the absorption at this wavelength. The linear increase of the absorbance measured at 688 nm, as a function of the number of bilayers, after a baseline correction, indicates the regular deposition of both components (Figure 9).

The measured absorbance was very near that observed for 100 nm—thick films of PANI/NiTsPc [46], indicating a thickness of approximately the same order of magnitude. The absorbance was higher than with PDDA, because the PANI absorbed in the entire spectrum. Thus, the absorbance at 688 nm resulted from contributions of the two components of the hybrid film. It was worth noting that, in PDDA, the maximum wavelength of CuTsPc was 623 nm [33], as a result of a different arrangement in the solid state. Indeed, it is well known that phthalocyanines can arrange in J- or H- aggregates, leading to a shift of the maximum wavelength towards higher or lower values [50,51].

#### 2.6.2. Gas Sensing

To ensure high repeatability, the devices were always used in the same order. The first experiment was a current-voltage (I-V) characteristic measured between −2 and +2 V, at 0.1 V·s^−1^ and recording one point every second, in order to characterize the starting state of the sample, which was stored in the dark at room temperature. To put the sensor into common conditions, an experiment was performed under humidity, varying from 70% RH to 10% RH by step of 20% RH, for 10 min per step. This experiment was very important in order to begin the last wanted experiment, the ammonia sensing, always in the same atmospheric conditions.

**Figure 10.**
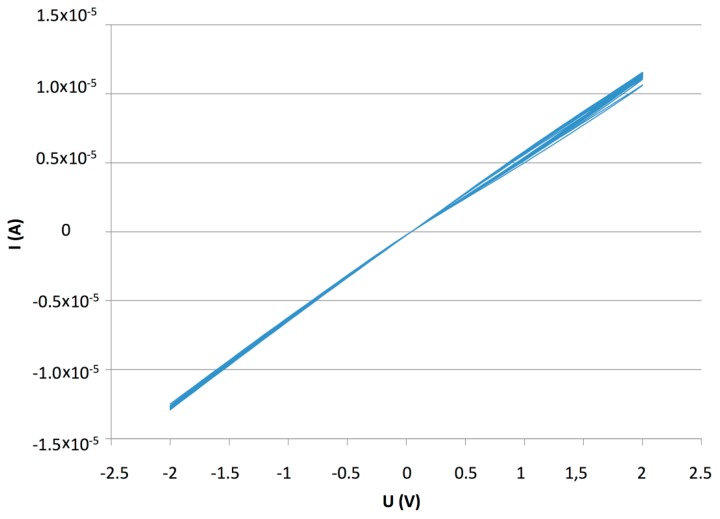
I-V characteristic of the (PANI/CuTsPc)_20_ device (E5), between −2 and +2 V.

**Figure 11.**
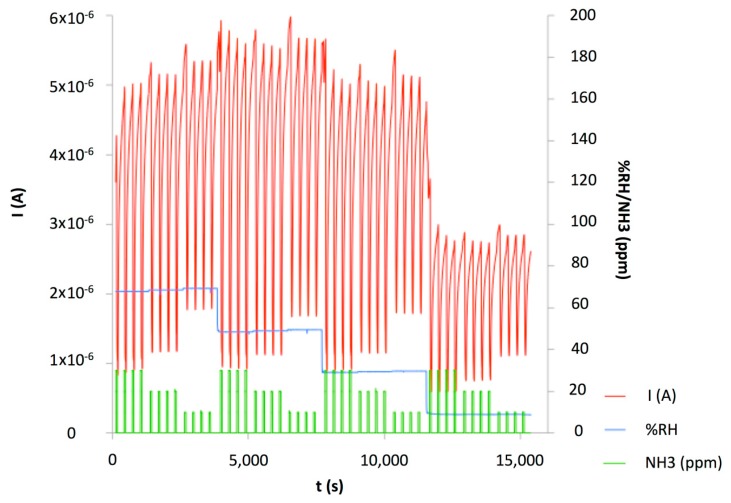
Ammonia/humidity exposure/recovery of a (PANI/CuTsPc)_20_ device (E5) at 30, 20 and 10 ppm ammonia, with 70% to 10% RH, polarized at 1 V.

The characteristic I-V plot (Figure 10), showed a high current compared to the PDDA/s-CoPc device, higher than 1.0 × 10^−5^ A, due to the highly conductive PANI. The humidity and humidity/ ammonia exposures were performed at +1 V. After the humidity sensing, humidity/ammonia experiments were performed using exposure/recovery cycles. The sensor was exposed from 30 to 10 ppm, four exposure/recovery cycles for each ammonia concentration, in wet atmosphere, from 70% to 10% RH under 500 mL·min^−1^ synthetic air flow (Figure 11).

**Figure 12.**
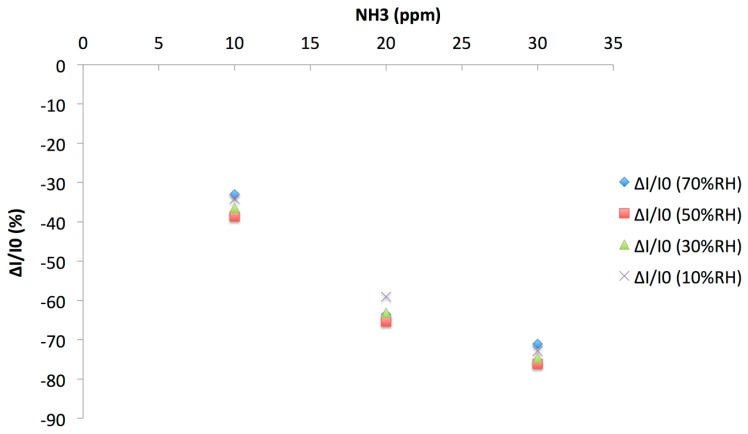
Relative response (ΔI/I_0_) of a (PANI/CuTsPc)_20_ device (E5) as a function of the NH_3_ concentration at various RH values, as calculated from four 0.25 min/1 min exposure/recovery cycles.

Ammonia exposure induced a decrease of the current measured, in accordance with the p-type nature of the majority charge carriers in the material. The response depends on the concentration: The lower the concentration, the lower the current decreased. We plotted the relative response ΔI/I_0_(%) = ((I − I_0_)/I_0_) × 100 as a function of the NH_3_ concentration at various RH values, as calculated from four 0.25 min/1 min exposure/recovery cycles (Figure 12).

It appeared clearly that the effect of RH, between 10% and 70%, was lower than the effect of tens of ppm of NH_3_. From this plot, the limit of detection (LOD) was determined, as 3 times the signal/noise ratio; at 50% RH it was 0.7 ppm, for a 0.25 min—long exposure period. It means that, with longer exposure periods, typically 1 min, the LOD would be still lower. The exposure duration could be adapted as a function of the target application. The sensor response was totally reversible. These results were comparable to those obtained previously [52], but the main difference was that the response to NH_3_ was studied at different RH values. This allowed us to study a possible synergy between NH_3_ and water effects. In [52], the response to humidity was studied without NH_3_ and *vice versa*, the sensitivity to NH_3_ was determined in dry air. It is worth noting that PANI-CuTsPc films prepared also during the present study by solvent cast were more sensitive to water, with poor NH_3_ sensing performance compared to the LBL-—deposited films.

**Figure 13.**
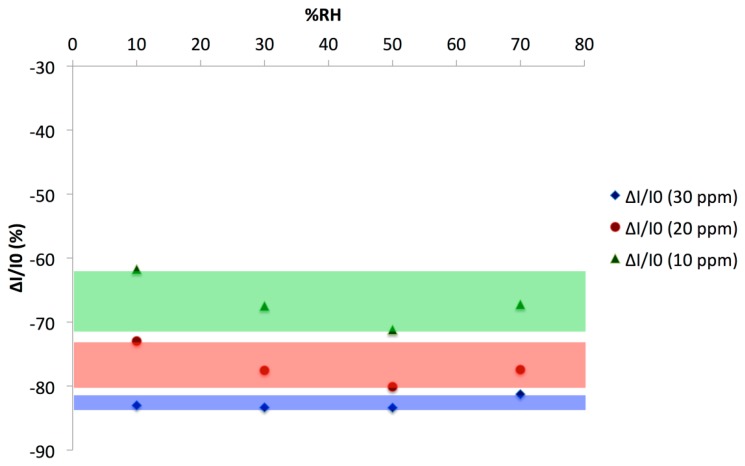
Relative response (ΔI/I_0_) of a (PANI/CuTsPc)_20_ device (E5) to ammonia against the Relative Humidity, as calculated from four 1 min/4 min exposure/recovery cycles. From bottom to top, the ammonia concentration is 30, 20 and 10 ppm. The green, red and blue bands show the range of the relative response for 10, 20 and 30 ppm, respectively.

**Figure 14.**
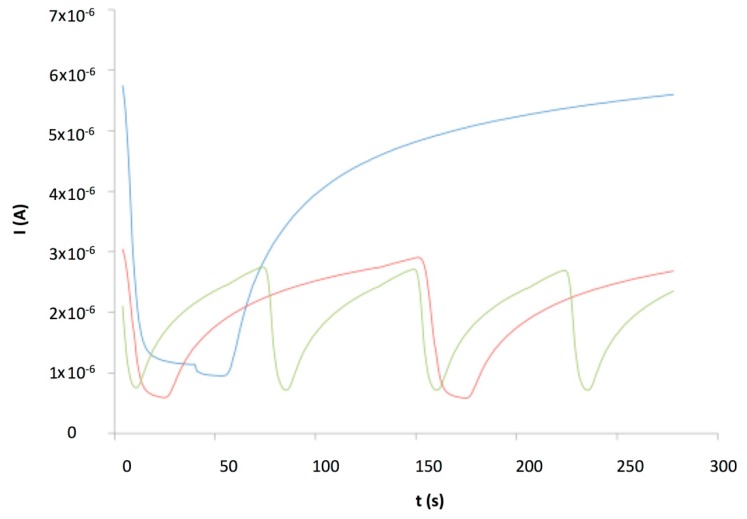
Current *versus* time of a (PANI/CuTsPc)_20_ device (E5) exposed to 30 ppm NH_3_ under 50% RH, with different exposure/recovery times, namely 1 min/4 min (**blue**), 0.5 min/2 min (**red**) and 0.25 min/1 min (**green**).

The relative response to NH_3_ was also plotted as a function of the RH value (Figure 13). The relative response varied from −61% at 10 ppm ammonia and 10% RH to a maximum of −83% at 30 ppm ammonia and 30 or 50% RH. The difference between the relative response at 20 ppm and 30 ppm was slightly higher than one unit (%), comparing the maximum at 20 ppm, −80%, and the minimum at 30 ppm, −81%. The current decreased and the saturation of the sensor appeared to be fast. This is the reason why the exposure/recovery protocol was adapted, from 1 min exposure and 4 min recovery down to 0.5 and 0.25 min exposure and 2 and 1 min recovery, respectively (Figure 14).

As we showed previously with an electrodeposited polypyrrole/phthalocyanine resistor [30], the effect of water was important at very low relative humidity, due to the hydrophilicity of the material. It is true that the water contain in a material as a function of the relative humidity in atmosphere usually exhibits an hysteresis [53]. It means that the absolute water contain in the sensing materials is not the same when the RH is increasing or decreasing. However, in our experimental conditions, the gas flow is oriented directly on the sensor surface, so it improves the kinetics of adsorption/desorption. Moreover, the exposure to a constant RH was very long, about 1 h, and at each RH value we could not see an important drift that could be due to a water contain evolution during these 1 h—long periods (see Figure 11). So, we believed that operating from low to high RH instead of from high to low RH values as we did would not affect the general behavior of the device.

This graph showed the saturation effect with 1 min (blue curve) and 0.5 min (red curve) exposure periods, but not for the 0.25 min exposure and 1 min recovery periods (green curve). This later should be the most efficient protocol and it was verified, comparing the relative response for each protocol to know if there is a real improvement of the discrimination between the different ammonia concentrations and the large humidity range (Table 1). From 0.25 min to 1 min exposure, the maximum relative response was reduced by only 7%.

The saturation effect was clearly deduced from the variation, in %, determined for each exposure/recovery condition (Table 1). These variations were calculated between the responses to two consecutive NH_3_ concentrations, from the minimum relative response to a NH_3_ concentration minus the maximum relative response of the immediately lower concentration. As example, with the 1 min/4 min exposure/recovery protocol, the maximum relative response at 20 ppm NH_3_, namely −80.11%, was observed at 50% RH, and for the same protocol at 30 ppm NH_3_, the minimum relative response, namely −81.26%, was seen at 70% RH. From those values and for these exposure/recovery cycles, the variation, in %, was determined to be −81.26%–(−80.11%) = −1.14%, which corresponded to the minimum difference between the relative responses to 20 and 30 ppm NH_3_ seen by the sensor, whatever was the relative humidity and without any information about it.

**Figure 15.**
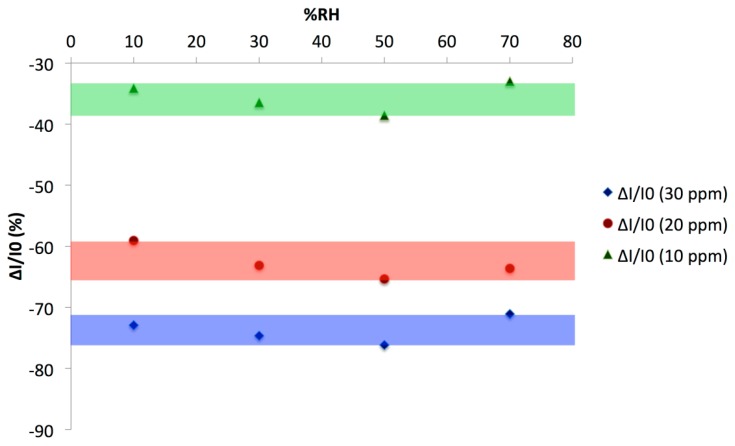
Relative response (ΔI/I_0_) of a (PANI/CuTsPc)_20_ device (E5) to NH_3_ as a function of the Relative Humidity, as calculated from four 0.25/1 min exposure/recovery cycles. From bottom to top, the NH_3_ concentration is 30, 20 and 10 ppm. The green, red and blue bands show the range of the relative response for respectively 10, 20 and 30 ppm NH_3_.

Simultaneously, we observed an increase by 5 times of the variation, in %, between 20 and 30 ppm NH_3_, from −1.14% to 5.70%, leading to an improved discrimination between the responses to the different ammonia concentrations. This discrimination can be clearly seen when plotting the relative response (ΔI/I_0_) to NH_3_ as a function of the RH value, as determined with the 0.25/1 min exposure/recovery protocol (Figure 15). On this figure, the improved discrimination between the ammonia concentrations was clearly visible compared to Figure 12, plotted at the same scale. The colored bands were well separated with the optimized protocol. With the 0.25 min exposure/1 min recovery optimized protocol, the sensor was able to discriminate NH_3_ concentrations by steps of 10 ppm, whatever the humidity value between 10% and 70%RH. This protocol has also the advantage to be 4 times faster, allowing 4 cycles instead of 1 in the same period.

## 3. Conclusions

In this study, two sulfonated phthalocyanines were combined with polymers of different nature. When associated with hydrophilic non-conductive polymers like PAA, PEG and PVP, the sulfonated phthalocyanine led to hybrid films, prepared by solvent-casting, that were highly sensitive to water. The layer-by-layer technique was suitable for the preparation of PDDA polycation/sulfonated phthalocyanine, with an increase of the absorbance of the film as a function of the number of layers. However, these films were too insulating to be used as sensing materials in conductometric sensors. On the contrary, polyaniline/tetrasulfonated copper phthalocyanine films, also prepared by the layer-by-layer technique from aqueous solutions, were suitable for gas sensing applications. The deposition was monitored by the absorbance that increased linearly as a function of the layer number. We showed that these hybrid materials were capable of operating as sensing materials for the detection of ammonia in a broad range of relative humidity. A relative response to 30 ppm NH_3_ as high as −83% was observed. In addition, we demonstrated that the response depended on the exposure duration. A 0.25 min/1 min exposure/recovery cycle led to a better discrimination of the ammonia concentration, by step of 10 ppm, by keeping the sensitivity better than −70%, no matter the relative humidity in the range 10%–70% RH range. Compared to both individual materials, the hybrid films exhibited a strongly enhanced sensitivity to ammonia and stability towards humidity.

## Figures and Tables

**Table 1.** Relative response (ΔI/I_0_ (%)) to NH_3_ depending on the relative humidity for the different exposure/recovery protocols. The variations (in %) between the responses to two consecutive NH_3_ concentrations were calculated for each exposure/recovery protocol, from the minimum relative response to a NH_3_ concentration minus the maximum relative response of the immediately lower concentration.

Exposure/Recovery	1 min/4 min			0.5 min/2 min			0.25 min/1 min		
RH (%)/NH_3_ (ppm)	10	20	30	10	20	30	10	20	30
70	−67.24	−77.46	−81.26	−51.40	−73.46	−78.51	−33.00	−63.64	−71.07
50	−71.21	−80.11	−83.39	−57.22	−74.86	−80.14	−38.58	−65.37	−76.15
30	−67.48	−77.56	−83.35	−54.85	−72.29	−79.27	−36.47	−63.15	−74.66
10	−61.76	−73.00	−83.04	−50.50	−67.75	−78.55	−34.13	−59.07	−72.90
Variation (%)		−1.79	−1.14		−10.53	−3.65		−20.50	−5.70
